# Semicontinuous Bioreactor Production of Recombinant Butyrylcholinesterase in Transgenic Rice Cell Suspension Cultures

**DOI:** 10.3389/fpls.2016.00412

**Published:** 2016-03-31

**Authors:** Jasmine M. Corbin, Bryce I. Hashimoto, Kalimuthu Karuppanan, Zachary R. Kyser, Liying Wu, Brian A. Roberts, Amy R. Noe, Raymond L. Rodriguez, Karen A. McDonald, Somen Nandi

**Affiliations:** ^1^Chemical Engineering and Materials Science, University of California, DavisDavis, CA, USA; ^2^Arcadia BiosciencesDavis, CA, USA; ^3^Leidos, Inc.Frederick, MD, USA; ^4^Global HealthShare®, Molecular and Cellular Biology, University of California, DavisDavis, CA, USA

**Keywords:** butyrylcholinesterase, rice amylase 3D, inducible promoter, plant cell culture, semicontinuous culture

## Abstract

An active and tetrameric form of recombinant butyrylcholinesterase (BChE), a large and complex human enzyme, was produced via semicontinuous operation in a transgenic rice cell suspension culture. After transformation of rice callus and screening of transformants, the cultures were scaled up from culture flask to a lab scale bioreactor. The bioreactor was operated through two phases each of growth and expression. The cells were able to produce BChE during both expression phases, with a maximum yield of 1.6 mg BChE/L of culture during the second expression phase. Cells successfully regrew during a 5-day growth phase. A combination of activity assays and Western blot analysis indicated production of an active and fully assembled tetramer of BChE.

## Introduction

Butyrylcholinesterase (BChE, EC 3.1.1.8) is a native human serine hydrolase enzyme that has been shown to function as a bioscavenger against various organophosphorus nerve agents, with both prophylactic and therapeutic applications (Lenz et al., 2005). BChE is a complex tetrameric protein comprised of four identical 85 kDa monomers, each with 9 N-linked glycosylation sites (Lockridge, 2015). BChE can be purified from expired human blood plasma, but low yields and high costs (anticipated >US$10,000 per treatment dose) limit its use (DARPA, 2012). Therefore, there is a pressing need for a cost-effective platform for recombinant production of BChE.

Recombinant BChE has been successfully produced in several expression systems, including transgenic goats and mice (Huang et al., 2007), insect cells (Brazzolotto et al., 2012), transgenic plants (Geyer et al., 2010), transient expression in plants (Schneider et al., 2014a), and CHO cells (Ilyushin et al., 2013; Terekhov et al., 2015). However, a major limitation in the production of recombinant BChE is the need to produce tetrameric BChE, which has a significantly longer circulatory half-life than the dimeric or monomeric forms (Duysen et al., 2002). Many of these systems show incomplete tetramerization of the molecule (Huang et al., 2007; Geyer et al., 2010; Brazzolotto et al., 2012; Schneider et al., 2014b). While there has been success with production of fully tetrameric BChE in CHO cells through coexpression of the enzyme with a proline-rich peptide (Terekhov et al., 2015), mammalian systems are susceptible to contamination with mammalian pathogens and require extensive regulatory clearances for human therapeutic use. Whole plant systems can avoid this problem, but also require specialized facilities to grow and harvest transgenic material or to transiently express foreign proteins.

Plant cell suspension cultures, however, offer many advantages over alternative expression systems for the production of human therapeutics. These include (1) a simple, low cost, animal component-free, chemically-defined medium; (2) lack of susceptibility to contamination with mammalian pathogens; and (3) the ability to perform complex post-translational modifications such as glycosylation (Huang and McDonald, 2012). Unlike with whole plant systems, plant cell cultures can make use of already existing cell culture infrastructure. Thus, a plant cell suspension culture is well suited to address the difficulties associated with production of BChE.

In particular, the use of the rice alpha amylase 3D (RAmy3D) expression system in a rice cell suspension culture enables efficient, high-level expression of foreign proteins (Huang et al., 2001). The RAmy3D system contains an inducible promoter that is activated by sugar starvation and a signal peptide that tags the protein for secretion into the culture medium. This allows for a cyclical or semicontinuous operation of the culture in which the cells are subjected to multiple phases of growth and expression by alternating between a sugar-rich and sugar-free medium. In addition, use of an inducible expression system may alleviate some of the problems associated with reduced productivity over long culture times that has been observed for plant cell cultures that utilize constitutive expression systems (Raven et al., 2015). Semicontinuous operation can further reduce culture costs by eliminating the shut down and start up time between runs that would be necessary in a batch culture system. Although RAmy3D has been successfully used for the production of other recombinant proteins in rice cell culture (Huang et al., 2002; Trexler et al., 2005; Park et al., 2010), it has not been used to produce a molecule as large and complex as BChE.

To address the need for effective, scalable, and active BChE, we have designed and studied a transgenic rice cell suspension culture for the production of BChE using the RAmy3D expression system. This study demonstrates semicontinuous bioreactor production of an active form of BChE, which with continued development, can provide a reliable system for production of this molecule as a therapeutic or prophylactic.

## Materials and methods

### Expression vector design and cloning

The native human BChE (NCBI NM_000055) coding sequence without the native secretion signal peptide was modified for expression in rice and inserted into a vector (pUC57) containing the RAmy3D promoter, signal peptide, and terminator sequences using GenScript (GenScript, Piscataway, NJ). The native human BChE coding sequence was codon optimized for expression in rice and analyzed using Visual Gene Developer (http://visualgenedeveloper.net). The sequence was modified without any changes in the amino acid sequence of the mature protein. The overall GC content of the DNA sequence was increased from 40.2 to 51.4%, and the codon adaptation index was changed from 0.68 to 0.83. The final construct was then cloned in a binary vector (pCAMBIA2300) and incorporated via heat shock into *Agrobacterium tumefaciens* strain EHA 105 for transformation.

### Transformation and selection of callus

The transformation was performed as described in Huang et al. (2001) with minor modifications. Callus derived from seeds of *Oryza sativa* cv. Taipei 309 were co-cultivated with *A. tumefaciens* containing the described vector at OD_600_ = 1.0 for 20 min. The callus was transferred to semi-solid medium and incubated in the dark at 25°C for 3 days before washing three times with a solution of 200 mg/L timentin and 250 mg/L cefotaxime. Finally, the callus was transferred to a sucrose (S) rich semi-solid selection medium (“NB”) that contains N6 macronutrients (Chih-Ching et al., 1975), B5 micronutrients and vitamins (Gamborg et al., 1968), 2 mg/L 2,4-dicholorophenoxyacetic acid (2,4-D), 250 mg/L L-proline, 250 mg/L L-glutamine, 300 mg/L casein hydrolysate, 30 g/L sucrose, 1.8 g/L gelzan, and 50 mg/L geneticin as the selection antibiotic, denoted as NB+S. Previous studies show that supplementation with proline and glutamine promotes callus induction (Pawar et al., 2015).

### Screening of transformed callus

Transformed callus was subjected to eight rounds of screening at increasingly larger scales in selection media containing geneticin (as the selection antibiotic). The first round of screening evaluated 310 transformation events and was performed in a 96 well plate. Callus was transferred from semi-solid NB+S to a well containing liquid NB+S (same composition as described above) and stored in an Innova 4000 incubator/shaker (Eppendorf, Inc., Hauppauge, NY) at 60 rpm and 27°C in the dark for 5 days. Induction was performed by sterilely pipetting off the spent NB+S medium and replacing it with an equal volume of NB−S medium, which has the same composition as NB+S except that the sucrose (S) is replaced with 8 g/L mannitol. Samples of both the spent medium and biomass were collected and analyzed at 120 h after induction. After the fourth round of screening, the top 10 transformation events were used to establish shake flask cultures. Flasks with nominal volumes between 125 mL and 1 L were filled to 1/5 full and maintained at 140 rpm and 27°C in the dark in the same incubator/shaker. Flask screenings were performed in the same manner as well plate screenings. After the 8th round of screening, a single, top-performing cell line was selected based on cell physiology (healthy appearance, light color, friable aggregates) and BChE expression stability, and suspension cultures of this line were maintained through weekly sub-culturing with fresh liquid NB+S medium.

### Bioreactor operation

Cultures were scaled up for operation in a 5 L bioreactor (BioFlo 3000, Eppendorf, Inc., Hauppauge, NY) equipped with a single pitched blade impeller. To obtain small and consistently sized aggregates, cultures were sieved at the time of inoculation by pressing the biomass through a 280 μm pore size stainless steel mesh sieve (Sigma Aldrich, St. Louis, MO).

The bioreactor was operated under conditions similar to those described in Trexler et al. (2002) with slight modifications. The bioreactor was maintained at 27°C, 75 rpm (agitation speed), and 40% (air saturation) dissolved oxygen (Mettler Toledo O_2_ sensor). The dissolved oxygen level in the culture was controlled by altering the concentration of oxygen, nitrogen, and air in the gas sparging stream, and the overall gas flow rate was maintained between 0.2 and 0.4 vvm. The oxygen uptake rate was determined by measuring the change in dissolved oxygen in the culture in the absence of aeration. The pH of the culture was monitored but not controlled. The cultures were grown under ambient light, and the conditions were identical for both growth and expression phases.

Induction of BChE expression in the bioreactor was performed using gravity sedimentation. After the biomass was allowed to settle, a peristaltic pump and sterile tube welder were used to remove the spent NB+S medium and replace it with fresh NB−S medium. For semicontinuous operation, another growth phase was initiated by following the same procedure described above, by removing the NB−S medium and replacing with NB+S medium.

### Biomass measurements

Four well-mixed 10 mL samples were taken every 24 h (48 h during the initial growth period) from the reactor under sterile conditions through the sampling port. One sample was set aside for protein extraction and quantification, and the remaining three were used to determine the fresh weight (FW) and dry weight (DW) of cells by washing them in 10 mL double distilled water (to remove residual sugars) on a pre-weighed 1.6 μm Binder-Free Glass Microfiber filter (Whatman GF/A 4.7 cm, GE Healthcare Life Sciences, Pittsburgh, PA). The combined filter and biomass was weighed immediately to obtain the FW, then dried in an oven at 65°C for 24 h and weighed again to obtain the DW.

### Sugar analysis

The concentrations of sucrose and glucose in the culture medium were measured using YSI 2900 Biochemistry Analyzer (Xylem, Inc., Rye Brook, NY). Cell-free medium from reactor sampling was passed through a 0.22 μm syringe filter to remove any remaining cells and stored at 4°C until analysis. The YSI 2900 enzymatically measures sucrose and glucose concentrations in a given sample and can account for any interaction between the signals from each. Samples were measured at 2X and 4X dilutions with double distilled water to ensure concentrations of each sugar were within the accurate detection range of the instrument.

### Quantification of active intracellular BChE

Concentration of active BChE was measured separately in the medium (by analyzing crude culture medium) and associated with the cell mass (by analyzing the crude cell extract). The cell extract was prepared by grinding the callus in a 1:1 ratio in cold homogenization buffer (100 mM sodium phosphate, 100 mM NaCl, pH 7.4) using a Tissue Tearor (Biospec Products, Bartlesville, OK) operated at maximum speed for 30 s. The homogenized sample was then centrifuged a 14,000 g for 10 min, and the supernatant was saved in a new tube and stored at 4°C until analysis.

Active BChE concentration in each sample was measured using a modified Ellman activity assay (Ellman et al., 1961). The assay monitors the hydrolysis of butyrylthiocholine in the presence of Ellman's reagent in 100 mM sodium phosphate buffer, pH 7.4. This reaction was monitored using a spectrophotometer (SpectraMax 340PC, Molecular Devices, Sunnyvale, CA) to measure the absorbance at 405 nm over a period of 3 min at 25°C. Samples were concentrated (with a 30 kDa ultrafiltration membrane) or diluted to fall within the accurate detection range of 0.5–1.0 ng BChE/μL sample. Once the concentration of BChE from a sample was determined, it was normalized to the mass of fresh weight biomass, volume of extraction buffer, or volume of total culture.

### Quantification of total soluble protein

The total soluble protein (TSP) in a sample was measured using a Bio-Rad Protein Assay kit (Bio-Rad, Hercules, CA), which is based on the method of Bradford (Bradford, 1976). The assay was performed per manufacturer's instructions. Each sample was analyzed in triplicate, including protein standards used to generate a standard curve.

### Gel electrophoresis and immunoblotting

SDS-PAGE was performed using a 4–15% gradient gel, and native-PAGE using a 7.5% gel (Mini-PROTEAN precast gels, BioRad, Hercules, CA). Western blotting was performed using a 1:200 dilution of mouse anti-BChE IgG as the primary antibody and a 1:2000 dilution of goat anti-mouse IgG-HRP as the secondary antibody (Santa Cruz Biotechnology, Dallas, TX), and developed by incubation with 3,3′,5,5′-tetramethylbenzidine (TMB) substrate. PEGylated recombinant human BChE derived from transgenic goats (PharmAthene, Inc, Annapolis, MD) and native equine serum BChE (Sigma Aldrich, St. Louis, MO) were used as controls.

## Results

### Transformation and screening

**Figure 1:**
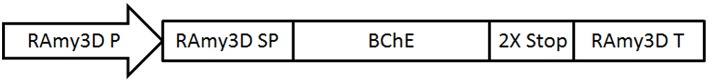
**A schematic representation of the expression vector for butyrylcholinesterase (BChE) under control of the rice alpha amylase 3D expression cassette**. RAmy3D P, rice alpha-amylase 3D promoter; RAmy3D SP, rice alpha-amylase signal peptide; BChE, codon optimized synthetic human BChE gene; RAmy3D T, rice alpha-amylase terminator.

The rice-optimized BChE gene construct was cloned into the RAmy3D (Huang et al., 2001) expression system (Figure 1) and subsequently into *A. tumefaciens* for gene delivery by co-cultivation. Stable integration of incoming recombinant DNA into cellular DNA is largely a random process, and accordingly, the sites of integration are dispersed throughout the genome. The ability to backcross and screen independent transgenic events can produce more stable arrangements and expression of the transgene in whole plants (Chih-Ching et al., 1975; Park et al., 2010). For transgenic cell lines, however, the process is more complex. Although primary calli can be selected for a particular antibiotic resistance and screen for the expression of a particular recombinant protein, genetic and epigenetic changes that occur during multiple cycles of cell differentiation and dedifferentiation can produce somaclonal variation in the calli (Gamborg et al., 1968). These somaclonal variants can exhibit a wide range of morphological and physiological phenotypes, including variations in the expression of the transgene.

**Figure 2:**
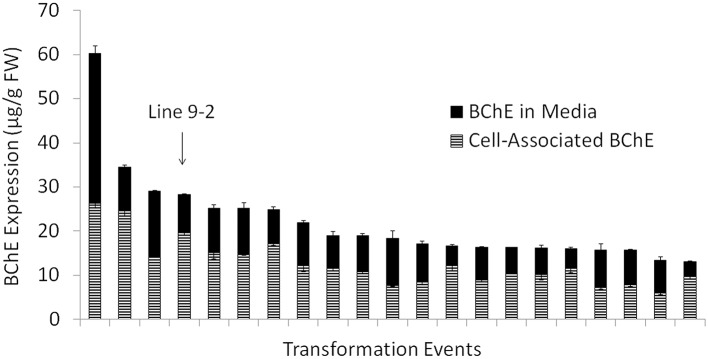
**Amount of active butyrylcholinesterase (BChE) normalized to the mass of fresh weight callus in the culture medium and associated with the biomass during the initial screening for cell line selection**. Data are shown for the 20 transformation events that produced highest amount of total active BChE out of 310 total transformation events screened. The top transformation event chosen for scale up and further study (line “9–2”) has been labeled.

To meet our goal for stable protein expression over many generations of microcalli propagation, extensive screening was performed to identify the lines that provide optimal protein production. Over 1000 transformants, from 310 independent transformation events were initially selected and grown on semi-solid selection medium. Of these, 105 events produced a detectable amount of BChE, with varying levels of expression (data not shown). Figure 2 shows the combined amounts of active secreted and cell-associated BChE from the top 20 transformation events during the first screening. After 8 rounds of screening, one cell line (identified as line “9–2”) was selected based on the expression stability and cell-line physiology for continued development and scale up, and was used for all remaining analyses described in this study.

### Cell growth, oxygen uptake, sugar consumption, and BChE expression kinetics

**Figure 3:**
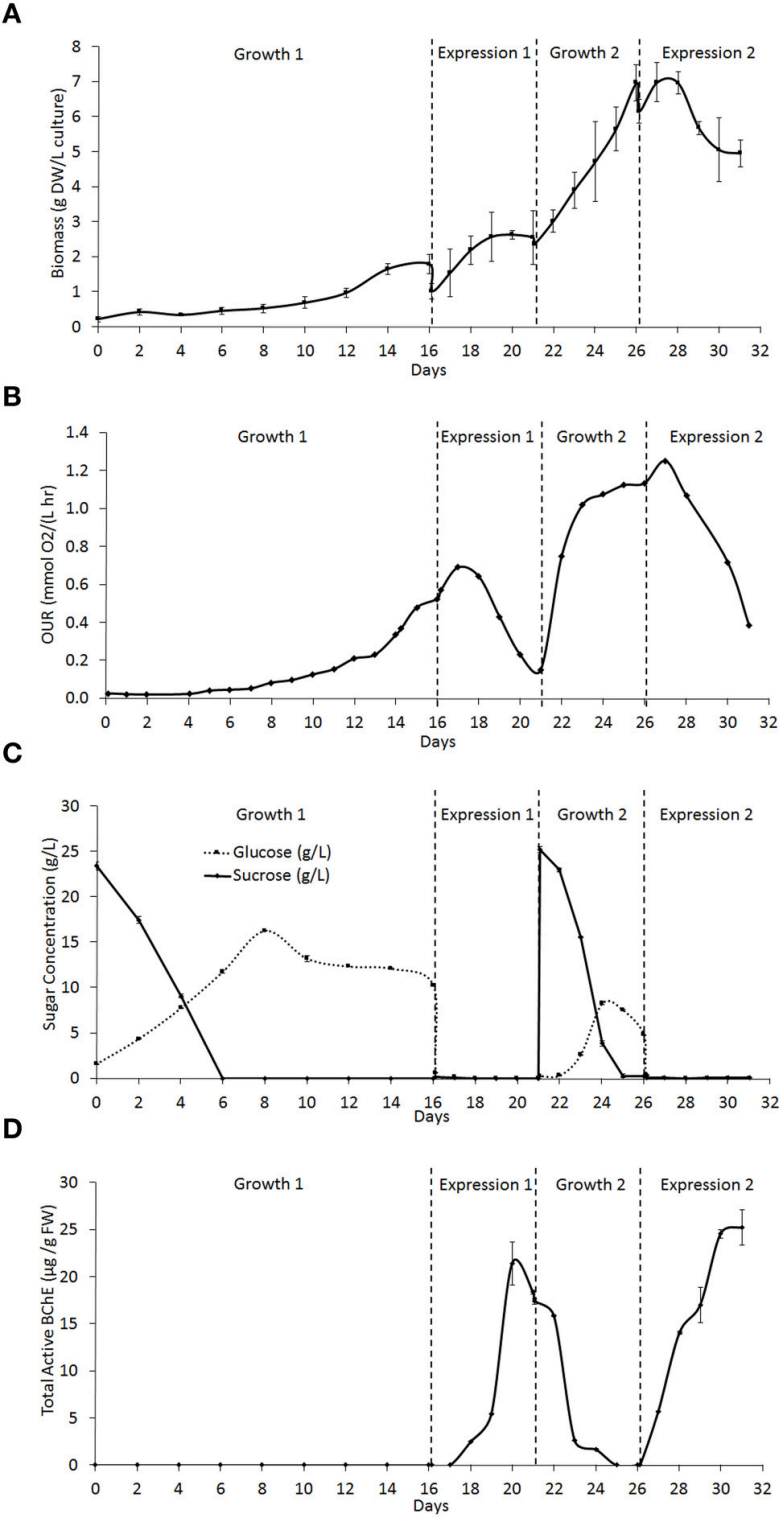
**Rice cell culture kinetics through two phases of cell growth and expression (as described in Section Materials and Methods) in a 5 L bioreactor. (A)** Concentration of biomass in the culture in g of dry weight (DW) callus per L of total culture. **(B)** Oxygen uptake rate (OUR) of the culture in mmol O_2_ per L of culture per hour. **(C)** Concentration of glucose and sucrose found in the culture medium in g/L. **(D)** Total active cell-associated butyrylcholinesterase (BChE) in μg, normalized to g of fresh weight (FW) callus.

The semicontinuous bioreactor culture was operated for 31 days and underwent two cycles each of growth and expression. Figure 3A shows the growth of the biomass over the duration of the culture. The first growth phase (Growth 1) had an initial biomass concentration of 0.21 ± 0.07 g DW/L of culture and lasted 16 days, while the second growth phase (Growth 2) had an initial biomass concentration of 2.37 ± 0.06 g DW/L and lasted 5 days. Growth 1 exhibited a long lag phase of 4 days followed by a long exponential phase of 12 days prior to induction, while Growth 2 had a lag of < 1 day and a 5 day exponential phase. This difference is likely due to the difference in the initial biomass concentration during each growth phase. During each expression phase, where NB+S was removed and replaced with NB−S, the biomass concentration immediately dropped as some of the biomass was pumped out of the reactor along with the spent medium. However, at the beginning of expression, the biomass concentration increased as the cells received fresh medium components such as nutrients and amino acids. The biomass concentration soon leveled off and began to drop as the cells starved from sugar deprivation, until a new growth phase was initiated. Table 1 summarizes the cell growth kinetics for both growth phases. The maximum specific growth rate (μ_max_) increased from 0.15 ± 0.01 day^−1^ during Growth 1 to 0.22 ± 0.01 day^−1^ during Growth 2, which correlated to a decrease in doubling time from 4.7 ± 0.3 to 3.2 ± 0.1 days.

**Table 1:** **Biomass growth and oxygen consumption kinetic parameters for two growth phases**.

**Phases**	**μ_*max*_(day^−1^)**	**τ_D_(days)**	**Maximum OUR [mmol O_2_/(L·h)]**	**Maximum specific OUR [mmol O_2_/(g DW·h)]**
Growth 1	0.15 ± 0.01	4.7 ± 0.34	0.524 ± 0.003	0.294 ± 0.046
Growth 2	0.22 ± 0.01	3.2 ± 0.12	1.251 ± 0.012	0.179 ± 0.015

*Key: μ_max_, maximum specific growth rate; τ_D_, doubling time; OUR, oxygen uptake rate; DW, dry weight of callus*.

The oxygen uptake rate (OUR) of the culture was also measured throughout operation as an indicator of the culture's metabolic activity (Figure 3B). A rise in OUR correlates with cell growth, and a drop correlates with the onset of the stationary phase. Our previous studies with *Nicotiana benthamiana* cell culture showed that the maximum value of OUR correlated with late exponential phase, and induction at this stage can lead to higher expression of a heterologous target protein (Huang et al., 2010). Thus, each expression phase was initiated as the OUR began to level off during the growth phase. However, the OUR reached a maximum 1 day after induction in both expression phases, and this may be explained in the same way as the initial rise in biomass concentration. After this initial rise, the OUR fell as the cells starved, until a new growth phase was initiated. Table 1 shows that maximum OUR increased from 0.52 mmol O_2_/(L·h) in Growth 1 to 1.25 mmol O_2_/(L·h) in Growth 2, which is expected because this value does not account for the increase in biomass density. The maximum specific OUR actually decreases from 0.29 mmol O_2_/(g DW·h) in Growth 1 to 0.18 mmol O_2_/(g DW·h) in Growth 2.

During growth phases, the sucrose concentration gradually decreased as it was hydrolyzed to produce equal concentrations of glucose and fructose, which can be consumed by the cells. Throughout the culture operation, the rate of hydrolysis of sucrose was faster than the rate of glucose consumption, which can be seen as a gradual decrease in sucrose concentration and increase in glucose concentration during both growth phases (Figure 3C). Both growth phases displayed the same trend, but Growth 2 showed the pattern on a shorter time scale, which is likely due to the increased biomass density. At the beginning of both expression phases, the sugar rich (+S) medium is removed and replaced with sugar free medium (−S), causing the concentration of both sugars to drop to and remain at 0 g/L for the duration of the expression phase.

Active BChE concentration rose gradually over time during each expression phase (Figure 3D). The first expression phase (Expression 1) produced a maximum active BChE concentration of 21.4 ± 2.3 *mu*g/g FW biomass, while the second expression phase (Expression 2) produced a maximum of 25.2 ± 1.9 *mu*g/g FW biomass. Expression 1 had a lag time between induction and expression of about 2 days, and reached its maximum expression level 4 days after induction. During Growth 2, the concentration of BChE decreased gradually until it was no longer detectable. Expression 2 had a lag of < 1 day, and reached a peak around 4 days after induction. These differences may be caused by differences in the timing of induction, the culture density, and the physiological condition of the cells. During both expression phases, the majority of BChE produced was associated with the cell mass. Negligible amounts of BChE were detected in the medium.

Table 2 summarizes the kinetic parameters of BChE expression. While the expression level on a per weight basis was similar for both expression phases (21.4 and 25.2 *mu*g/g FW), the increased biomass density during Expression 2 lead to a much higher amount of BChE produced per liter of culture (an increase from 0.72 mg/L culture during Expression 1 to 1.64 mg/L in Expression 2). Expression 2 also had a much higher maximum volumetric productivity (based on the cycle duration including growth and expression phases) due to the absence of a long growth lag phase and increased biomass density in Growth 2 as compared to Growth 1. Finally, the ratio of BChE to TSP increased from the first to second expression phase; this may be due to improved adaptation during subsequent induction cycles which resulted in increased expression of BChE.

**Table 2:** **Maximum values of butyrylcholinesterase (BChE) expression kinetic parameters for two expression phases**.

**Phases**	**BChE expression (*mu*g/g FW)**	**BChE expression (mg/L culture)**	**Volumetric productivity (*mu*g/L·day)**	**Purity (% BChE/TSP)**
Expression 1	21.4 ± 2.3	0.72 ± 0.16	35.8 ± 7.7	0.27 ± 0.02
Expression 2	25.2 ± 1.9	1.64 ± 0.15	183.8 ± 17.3	0.55 ± 0.03

*Key: FW, fresh weight of callus; TSP, total soluble protein*.

### Electrophoresis and immunoblotting

**Figure 4:**
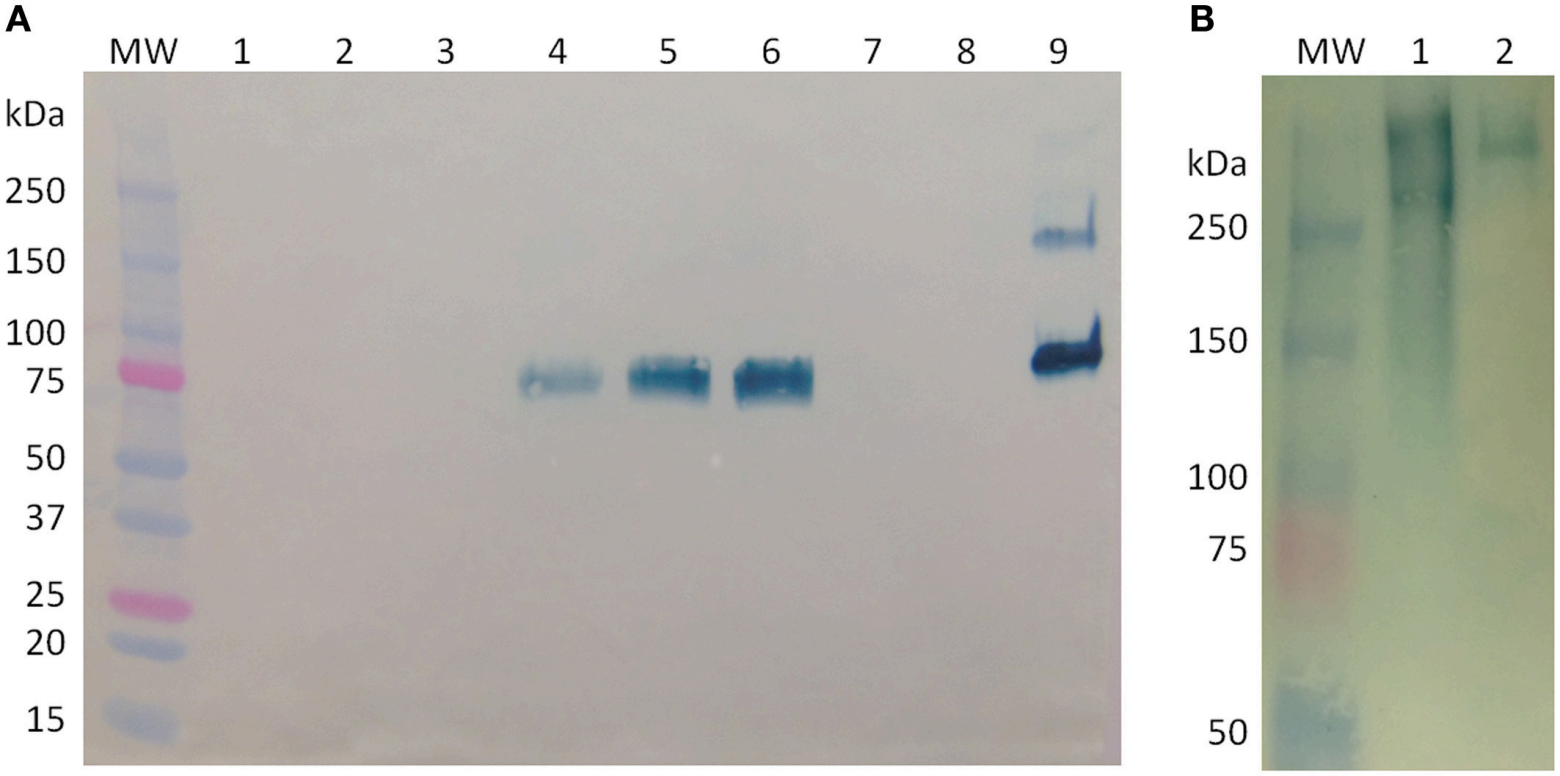
**Western blot analysis of butyrylcholinesterase (BChE) produced in rice cell culture. (A)** Western blot under reducing conditions of cell-associated samples from before and after medium exchanges. Each lane contains 20 *mu*L of crude cell extract. Lane MW: molecular weight ladder; lane 1, day 16, immediately before start of Expression phase 1; lane 2, day 16, immediately after start of Expression Phase 1; lane 3, day 18; lane 4, day 20; lane 5, day 21, immediately before start of Growth phase 2; lane 6, day 21, immediately after start of Growth phase 2; lane 7, day 23; lane 8, day 25; lane 9, control, 900 ng purified equine BChE. **(B)** Western blot under native conditions of BChE. Lane MW: molecular weight ladder; lane 1: 52 U (~200 ng) active BChE from an intracellular sample from day 3 of Expression phase 1; lane 2: control, 200 ng purified equine BChE.

Figure 4A shows a Western blot under reducing conditions of the cell-associated BChE from samples obtained during Expression 1 and Growth 2. Equal volumes (20 *mu*L) of crude cell extract were loaded into each lane. These extracts were obtained by grinding cells in a 1:1 ratio of biomass to buffer, so the intensity of the BChE band corresponds to the concentration of BChE associated with the cells at the time each sample was taken. Under reducing conditions, a distinct band is seen around 85 kDa, which corresponds to the predicted size of a monomer of BChE. Samples taken immediately before and after induction and 2 days after induction (lanes 1–3) show no visible band, which corresponds to the low BChE activity as seen in the activity data shown in Figure 3D (< 5 *mu*g/g FW). The band in lane 4 corresponds to a higher value of BChE activity (21 *mu*g/g FW). However, while we see a decrease in BChE activity from day 20 to 21 (a drop from 21 to 18 *mu*g/g FW), there is an increase in the intensity of the BChE band seen in the Western blot in lanes 5 and 6 (which correspond to samples taken immediately before and after initiation of Growth 2 on day 21). This may indicate that a portion of the BChE detected in the Western blot is inactive.

To determine the oligomerization status of the rice cell culture-produced BChE, we performed Western blot analysis under native conditions on a crude cell extract from Expression 1 (Figure 4B). Lane 2 contains 200 ng of purified equine serum BChE (as human BChE has been commercially unavailable for over a year), which is about 440 kDa in its tetrameric form. The cell extract shows two bands, one smaller and one larger than the equine control. The smaller product is near the expected size of tetrameric BChE (340 kDa), while the other may be an aggregation product.

## Discussion

The size and complexity of BChE has been a major impediment in its production in any recombinant expression host. Despite high expression levels (up to 5 g/L of milk), transgenic goats are able to produce primarily dimeric BChE, which exhibits a reduced circulatory half-life (~2 min) *in vivo* (Huang et al., 2007). Many attempts have been made to solve this problem, including PEGylation (Sun et al., 2013) or sialylation (Ilyushin et al., 2013) to increase this half-life, and addition of a proline-rich peptide to encourage tetramerization (Larson et al., 2014; Terekhov et al., 2015). Recent work by Schneider et al. also reports difficulty in secretion of BChE oligomers produced by transient expression in *N. benthamiana*, despite the molecule's ability to tetramerize in a crude cell extract (Schneider et al., 2014b). Recombinant tetrameric BChE has been produced in other cell culture types and reached up to 70 mg/L of culture (Terekhov et al., 2015), but these cultures have been limited by a comparatively high cost of culture operation and increased potential for contamination from human pathogens.

Previous work by our group has demonstrated successful semicontinuous production of a fully active alpha-1-antitrypsin under control of the RAmy3D expression system in a transgenic rice cell culture (Huang et al., 2001; Trexler et al., 2002, 2005). In the current study, we successfully produced an intact and active form of BChE, a larger and more complex glycoprotein therapeutic. A maximum of 1.6 mg/L of culture was produced during the second expression phase in a relatively non-optimized system. We expect that further refinements of the operational strategy will increase expression levels to those of other cell culture systems. For example, Park et al. have demonstrated a fed-batch operational strategy, in which the culture is supplemented with a concentrated solution of amino acids prior to sugar depletion, that can enhanced product yields up to 1.8-fold compared to induction via medium exchange (Park et al., 2010).

One major advantage of our system is that we can produce predominantly active and fully assembled tetrameric BChE. This underscores the importance of this system as a safe and cost-effective method for production of human biotherapeutics. In addition to growing on an inexpensive culture medium, long-term semicontinuous operation of the culture reduces the need for long seed trains and minimizes turn-around time, clean-in-place and steam-in-place operations, chemicals, and energy. Furthermore, the regulatory pathway for plant-based recombinant biologics for human therapeutic use has now been established with the production and regulatory approval of taliglucerase alfa (Elelyso^TM^) produced in carrot cell suspension in batch culture for the treatment of Gaucher's disease (Maxmen, 2012).

After the initial inoculation, the culture experienced a long lag phase in cell growth. The process of sieving the callus through a stainless steel mesh to obtain small cell aggregates may be physically stressful for the cells, thus requiring a longer initial growth period to acclimatize. This may be part of the reason that Growth 2, which involved the same biomass but no sieving immediately before the growth phase, did not exhibit the same lag phase and reached a maximum OUR value within 5 days. During expression phases, the culture reached a maximum expression level of BChE around day 4 after induction. Thus, after an initial acclimatization period, the culture can operated by alternating between 5-day growth phases and 4-day expression phases to maximize BChE productivity over longer periods.

The negligible release of BChE in the culture medium during bioreactor operation (in comparison to the screening stage, where a significant portion of BChE was secreted into the medium) may be due to the size of both the protein (340 kDa) and the larger size of the cell aggregates in comparison to the screening stage. For BChE to be released into the medium, BChE must not only be secreted from the cell, but must also pass through the cell wall and diffuse through the cell aggregate. The tetrameric form of BChE is about 50–60 nm (Lockridge et al., 2011), while the average pore size of the plant cell wall is 35–50 nm (Carpita et al., 1979) in differentiated plant cells. If tetrameric BChE is produced inside the cell and secreted in its fully assembled form, the protein may be trapped inside the cell wall or within the aggregate and only released during homogenization. Even if the tetramer is assembled after secretion, the monomers or the fully assembled tetramer still must diffuse through the cell aggregate to reach the culture medium. Because early screening experiments were done at small scale, the aggregates could be easily broken apart before expression level studies. Here, as shown in Figure 2, we saw roughly 30–60% of the total BChE secreted into the medium for most cell lines. However, with each subsequent screening, we saw a reduction in the proportion of BChE secreted into the medium. This can be explained by the fact that each subsequent screening was performed at an increasingly larger scale, which made it more difficult to break apart large aggregates. These larger aggregates provide a more challenging route through which secreted BChE must diffuse into the culture medium, and can explain why the proportion of secreted BChE dropped to nearly zero in later cultures.

The issue of low secretion levels may be addressed by decreasing the size of cell aggregates through alteration of medium composition or operating parameters (particularly, agitation, aeration rates, and length of growth and expression phases). However, it is also possible that oligomeric BChE is not able to correctly pass through the secretory pathway completely, as has been seen in other plant systems (Schneider et al., 2014b), and early screenings detected a small amount of monomeric BChE that was able to secrete effectively. To address this possibility, we are developing an alternate semicontinuous operation format similar to that described by Huang et al. (2010), in which a portion of the biomass is harvested after an expression phase, while the remaining biomass remains in the reactor as inoculum for the next growth phase.

## Conclusions and future prospects

Active recombinant BChE was produced in a transgenic rice cell suspension culture using an inducible gene expression system. Two complete phases of cell growth and BChE expression were performed, and the cells were able to express milligram quantities of active BChE during both expression phases and successfully regrow during a growth phase. Theoretically, these cultures could be operated indefinitely, and our goal is to evaluate the behavior and productivity of the culture during long-term (several months) operation, and if necessary by keeping a portion of the cells as inoculum to the medium in subsequent cycles. Further development of the culture and operation modes will aim to increase the amount of BChE produced and secreted into the culture medium, and to perform a more comprehensive characterization of BChE, including determination of the serum half-life and the glycosylation profile of the product.

## Author contributions

JC, led and designed experiments and wrote and edited the manuscript. BH, led and designed experiments. KK, conceptualized and assisted with performing and designing experiments. ZK, assisted with experiments. LW, performed experiments. BR, conceptualized. AN, conceptualized. RR, conceptualized and edited the manuscript. KM, conceptualized, designed experiments, and edited the manuscript. SN, conceptualized, designed experiments, and edited the manuscript.

### Conflict of interest statement

The authors declare that the research was conducted in the absence of any commercial or financial relationships that could be construed as a potential conflict of interest. KM is a co-founder of Inserogen, Inc., a plant-based biotechnology company with a focus on the development of orphan drugs for replacement therapy.

## References

[B1] Bradford M. M. (1976). A rapid and sensitive method for the quantitation of microgram quantities of protein utilizing the principle of protein-dye binding. Anal. Biochem. 72, 248–254. 10.1016/0003-2697(76)90527-3 942051

[B2] Brazzolotto X. Wandhammer M. Ronco C. Trovaslet M. Jean L. Lockridge O.. (2012). Human butyrylcholinesterase produced in insect cells: huprine-based affinity purification and crystal structure. FEBS J. 279, 2905–2916. 10.1111/j.1742-4658.2012.08672.x 22726956

[B3] Carpita N. Sabularse D. Montezinos D. Delmer D. P. (1979). Determination of the pore size of cell walls of living plant cells. Science 205, 1144–1147. 10.1126/science.205.4411.1144 17735052

[B4] Chih-Ching C. Ching-Chu W. Ching-San S. Chen H. Kwang-Chu Y. Chih-Yin C. (1975). Establishment of an efficient medium for anther culture of rice through comparative experiments on the nitrogen sources. Sci. China Math. 18, 659–668.

[B5] DARPA (2012). Broad Agency Annoucement 12-31: Butyrylcholinesterase Expression in Plants. Arlington, TX: D.S.O. Defense Advanced Research Projects Agency (DARPA).

[B6] Duysen E. G. Bartels C. F. Lockridge O. (2002). Wild-type and A328W mutant human butyrylcholinesterase tetramers expressed in chinese hamster ovary cells have a 16-hour half-life in the circulation and protect mice from cocaine toxicity. J. Pharmacol. Exp. Ther. 302, 751–758. 10.1124/jpet.102.033746 12130740

[B7] Ellman G. L. Courtney K. D. Andres V. Jr. Featherstone R. M. (1961). A new and rapid colorimetric determination of acetylcholinesterase activity. Biochem. Pharmacol. 7, 88–95. 10.1016/0006-2952(61)90145-9 13726518

[B8] Gamborg O. L. Miller R. A. Ojima K. (1968). Nutrient requirements of suspension cultures of soybean root cells. Exp. Cell Res. 50, 151–158. 10.1016/0014-4827(68)90403-5 5650857

[B9] Geyer B. C. Kannan L. Cherni I. Woods R. R. Soreq H. Mor T. S. (2010). Transgenic plants as a source for the bioscavenging enzyme, human butyrylcholinesterase. Plant Biotechnol. J. 8, 873–886. 10.1111/j.1467-7652.2010.00515.x 20353404

[B10] Huang J. Wu L. Yalda D. Adkins Y. Kelleher S. Crane M.. (2002). Expression of functional recombinant human lysozyme in transgenic rice cell culture. Transgenic Res. 11, 229–239. 10.1023/A:1015663706259 12113455

[B11] Huang J. M. Sutliff T. D. Wu L. Y. Nandi S. Benge K. Terashima M.. (2001). Expression and purification of functional human alpha-1-antitrypsin from cultured plant cells. Biotechnol. Prog. 17, 126–133. 10.1021/bp0001516 11170490

[B12] Huang K. McDonald K. A. (2012). Bioreactor systems for *in vitro* production of foreign proteins using plant cell cultures. Biotechnol. Adv. 30, 398–409. 10.1016/j.biotechadv.2011.07.016 21846499

[B13] Huang T. K. Plesha M. A. McDonald K. A. (2010). Semicontinuous bioreactor production of a recombinant human therapeutic protein using a chemically inducible viral amplicon expression system in transgenic plant cell suspension cultures. Biotechnol. Bioeng. 106, 408–421. 10.1002/bit.22713 20198659

[B14] Huang Y. J. Huang Y. Baldassarre H. Wang B. Lazaris A. Leduc M.. (2007). Recombinant human butyrylcholinesterase from milk of transgenic animals to protect against organophosphate poisoning. Proc. Natl. Acad. Sci. U.S.A. 104, 13603–13608. 10.1073/pnas.0702756104 17660298 PMC1934339

[B15] Ilyushin D. G. Smirnov I. V. Belogurov A. A. Jr. Dyachenko I. A. Zharmukhamedova T. Novozhilova T. I.. (2013). Chemical polysialylation of human recombinant butyrylcholinesterase delivers a long-acting bioscavenger for nerve agents *in vivo*. Proc. Natl. Acad. Sci. U.S.A. 110, 1243–1248. 10.1073/pnas.1211118110 23297221 PMC3557082

[B16] Larson M. A. Lockridge O. Hinrichs S. H. (2014). Polyproline promotes tetramerization of recombinant human butyrylcholinesterase. *Biochem*. J. 462, 329–335. 10.1042/BJ20140421 24916051

[B17] Lenz D. E. Maxwell D. M. Koplovitz I. Clark C. R. Capacio B. R. Cerasoli D. M.. (2005). Protection against soman or VX poisoning by human butyrylcholinesterase in guinea pigs and cynomolgus monkeys. Chem. Biol. Interact. 157–158, 205–210. 10.1016/j.cbi.2005.10.025 16289064

[B18] Lockridge O. (2015). Review of human butyrylcholinesterase structure, function, genetic variants, history of use in the clinic, and potential therapeutic uses. Pharmacol. Ther. 148, 34–46. 10.1016/j.pharmthera.2014.11.011 25448037

[B19] Lockridge O. Duysen E. G. Masson P. (2011). Butyrylcholinesterase: overview, structure, and function, anticholinesterase pesticides, in Anticholinesterase Pesticides: Metabolism, Neurotoxicity, and Epidemiology, eds Satoh T. Gupta R. C. (Hoboken, NJ: John Wiley & Sons, Inc.), 25–41.

[B20] Maxmen A. (2012). Drug-making plant blooms. Nature 485, 160. 10.1038/485160a 22575938

[B21] Park C.-I. Lee S.-J. Kang S.-H. Jung H.-S. Kim D.-I. Lim S.-M. (2010). Fed-batch cultivation of transgenic rice cells for the production of hCTLA4Ig using concentrated amino acids. Process Biochem. 45, 67–74. 10.1016/j.procbio.2009.08.004

[B22] Pawar B. Kale P. Bahurupe J. Jadhav A. Kale A. Pawar S. (2015). Proline and glutamine improve *in vitro* callus induction and subsequent shooting in rice. Rice Sci. 22, 283–289. 10.1016/j.rsci.2015.11.001

[B23] Raven N. Rasche S. Kuehn C. Anderlei T. Klockner W. Schuster F.. (2015). Scaled-up manufacturing of recombinant antibodies produced by plant cells in a 200-l orbitally-shaken disposable bioreactor. Biotechnol. Bioeng. 112, 308–321. 10.1002/bit.25352 25117428

[B24] Schneider J. D. Castilho A. Neumann L. Altmann F. Loos A. Kannan L.. (2014a). Expression of human butyrylcholinesterase with an engineered glycosylation profile resembling the plasma-derived orthologue. Biotechnol. J. 9, 501–510. 10.1002/biot.201300229 24130173 PMC3975692

[B25] Schneider J. D. Marillonnet S. Castilho A. Gruber C. Werner S. Mach L.. (2014b). Oligomerization status influences subcellular deposition and glycosylation of recombinant butyrylcholinesterase in *Nicotiana benthamiana*. Plant Biotechnol. J. 12, 832–839. 10.1111/pbi.12184 24618259 PMC4265266

[B26] Sun W. Luo C. Tipparaju P. Doctor B. P. Saxena A. (2013). Effect of polyethylene glycol conjugation on the circulatory stability of plasma-derived human butyrylcholinesterase in mice. Chem. Biol. Interact. 203, 172–176. 10.1016/j.cbi.2012.11.021 23220586

[B27] Terekhov S. Smirnov I. Bobik T. Shamborant O. Zenkova M. Chernolovskaya E.. (2015). A novel expression cassette delivers efficient production of exclusively tetrameric human butyrylcholinesterase with improved pharmacokinetics for protection against organophosphate poisoning. Biochimie 118, 51–59. 10.1016/j.biochi.2015.07.028 26239905

[B28] Trexler M. M. McDonald K. A. Jackman A. P. (2002). Bioreactor production of human *alpha*1-antitrypsin using metabolically regulated plant cell cultures. Biotechnol. Prog. 18, 501–508. 10.1021/bp020299k 12052066

[B29] Trexler M. M. McDonald K. A. Jackman A. P. (2005). A cyclical semicontinuous process for production of human *alpha*1-antitrypsin using metabolically induced plant cell suspension cultures. Biotechnol. Prog. 21, 321–328. 10.1021/bp0498692 15801766

